# Cold and hot consumption and health outcomes among US Asian and White populations

**DOI:** 10.1017/S000711452510514X

**Published:** 2025-10-28

**Authors:** Tianying Wu, Neeraja Ramesh, Cassie Doyle, Fang-Chi Hsu

**Affiliations:** 1 Division of Epidemiology and Biostatistics, School of Public Health, San Diego State University, San Diego, CA 92182, USA; 2 Moores Cancer Center, School of Medicine, University of California, San Diego, CA 92037, USA; 3 Department of Pediatrics, University of California of San Diego, San Diego, CA 92093, USA; 4 Department of Biostatistics and Data Science, Division of Public Health Sciences, Wake Forest University School of Medicine, Winston-Salem, NC 27101, USA

**Keywords:** Cold stress, Mental health, Sleep, Gut health

## Abstract

This study examined the associations between cold and hot food and beverage consumption and various health outcomes among Asians and Whites in the USA. Data were drawn from 212 Asian and 203 White adults (aged 18–65 years) in the Healthy Ageing Survey. Participants reported their frequency of cold and hot drink and meal intake, along with symptoms of depression, anxiety, insomnia and gastrointestinal issues (e.g. gas, abdominal fullness). Multivariable analyses adjusted for confounders were used to assess these associations. Among Asians, higher cold consumption in summer was associated with increased anxiety (*β* = 0·24, 95 % CI: 0·05, 0·44) and abdominal fullness (*β* = 0·05, 95 % CI: 0·01, 0·86). In contrast, among Whites, higher winter hot drink intake was linked to lower insomnia (*β* = –0·23, 95 % CI: –0·42, –0·04) and gas symptoms (*β* = –0·05, 95 % CI: –0·09, –0·01). Tertile analyses showed that, compared with tertile 1, Asians in the highest tertile of summer cold drink intake had higher insomnia scores (*β* = 1·26, 95 % CI: 0·19, 2·33), while Whites in the highest tertile of winter hot drink intake had lower depression scores (*β* = –1·73, 95 % CI: –3·28, –0·18). These associations were stronger among individuals with cold hands but not observed in those without. Findings suggest that the temperature of foods and beverages may influence mental and gut health, underscoring the need to consider temperature-related dietary habits in public health and nutrition strategies, particularly across diverse populations.

Asia accounts for 60 % of the global population,^(1)^ and Asians are the fastest-growing immigrant group in the USA^(2)^. Many Asian immigrants in the USA undergo acculturation, reflected in changes like language use and dietary habits^(3–5)^. However, little research has examined their dietary patterns and related health effects, particularly regarding beverage and food temperature.

In traditional Asian cultures, especially Chinese, consuming cold drinks and foods is seen as unhealthy, while warm or hot drinks and foods are considered harmless or even beneficial – especially in winter – a view rooted in traditional Chinese medicine^(6,7)^. Similar beliefs are found in Indian Ayurvedic practices, influenced by shared philosophies^(8)^. These views may vary by different Asian regions, shaped by exposure to traditional Chinese medicine or comparable traditions. Understanding such practices in Asians as a whole, as well as specific subgroups, is essential for evaluating their potential health implications.

White Americans constitute the majority of the USA population, and avoiding cold drinks and foods is generally not emphasised in Western culture, particularly in the USA. Evaluating the effects of cold and hot drink and food consumption in this group may help determine whether this traditional Asian practice is culturally specific or has broader health implications for non-Asian populations.

Although limited human studies have examined the health effects of cold drinks and foods, some evidence suggests cold beverages can disrupt gut microbiota in animals^(9,10)^ and lower core body temperature in elderly adults^(11)^. Disrupted microbiota and reduced core temperature are linked to various health concerns, including impaired gut circulation, and mental health issues^(12–14)^. Conversely, heating therapy, drinking warm or hot water and warm water irrigation during surgery have been shown to improve blood circulation, alleviate gastrointestinal (GI) symptoms in patients and help maintain core body temperature during surgical procedures^(15–17)^. Our group recently published the first study linking cold drink and ice cream consumption to dysmenorrhea^(18)^ – a condition associated with vascular disease and irritable bowel syndrome^(19–21)^. These findings underscore the need to explore cold and hot consumption patterns, their health impacts and differences between Asian and White populations. Given the greater prevalence of cold drink and food consumption in the USA compared with Asian countries, investigating this issue is especially urgent.

The aim of this study is to examine cold and hot food and beverage consumption among Asians and Whites and their associations with multiple health outcomes, including self-reported mental health (depression and anxiety symptoms), insomnia symptoms – which are associated with mental health – and gut health (i.e. sensations of abdominal fullness and gas).

## Methods

### Study design and population

The Asian and White population included in this study was selected from the Healthy Ageing Survey, launched by our research group in January 2022 to identify novel ageing-related risk factors in the general population, with a particular focus on disparities between Asians and non-Asians, as limited research has been conducted to examine the disparities between Asian and other ethnic groups. This study was conducted in accordance with the guidelines outlined in the Declaration of Helsinki. All procedures involving human subjects/patients were approved by the specific ethics committee, the Institutional Review Board of San Diego State University (ethics number: HS-2022-0010). Written informed consent was obtained from all participants. We included all racial groups, both men and women, aged 18–65 years, with at least 50 % of the total population identifying as Asian. To mitigate the influence of specific infectious diseases, we excluded individuals with HIV, hepatitis and active tuberculosis.

Recruiting the Asian population required extra effort, as Asians account for less than 10 % of the USA population, although this proportion is increasing rapidly^(22)^. The details were documented in a previous paper^(18)^. Briefly, we leveraged both local and national resources by recruiting Asians locally through multiple avenues, such as community outreach, local organisations, alumni networks and various Asian-dense communities in San Diego. We also recruited participants from other parts of Southern and Northern California, as well as other states, through Asian registries such as the Collaborative Approach for Asian Americans, Native Hawaiians and Pacific Islanders Research and Education, general research registries like ResearchMatch and Asian organisations, including Asian Pacific Islander Desi American and Alliance for Impact. Additionally, we utilised social media platforms, such as WeChat, to reach Chinese participants. These comprehensive recruitment strategies enabled us to effectively engage and recruit participants from diverse Asian backgrounds.

Asians and Whites selected for this study completed questionnaires on the frequency of consuming hot and cold drinks and meals during winter and summer, as well as on proposed health outcomes, including symptoms of depression, anxiety, insomnia and GI symptoms such as sensations of abdominal fullness and gas. A total of 212 Asians and 203 Whites were included in the study. Black, Hispanic and other ethnic groups were not included due to small sample sizes, which were unlikely to yield meaningful statistical results.

### Assessment of cold and hot consumption

This study is based on a cross-sectional survey. We assessed both the temperature preferences of drinks and food and the associated health outcomes within a single survey. For hot and cold consumption, participants were asked about their intake during both summer and winter over the past 12 months.

#### Assessment of temperature preferences for water and drinks during winter and summer

We asked participants about the frequency of consuming cold and hot water or drinks (e.g. milk, juice and tea) separately. Cold drinks were defined as water or drinks at approximately 4°C (39·2°F), including those taken directly from the refrigerator or served iced. Hot drinks were defined as water or other beverages served above room temperature, typically ≥ 40°C (104°F), such as hot coffee or tea. Participants reported their intake of cold and hot water or drinks during both summer and winter over the past 12 months. They selected from the following multiple-choice responses: Never or less than once per month, 1–3 times per month, once per week, 2–4 times per week, 5–6 times per week, 1–2 times per day, 3–5 times per day or 6+ times per day.

#### Assessment of meal temperatures during winter and summer

We asked participants about the frequency of consuming entirely or mostly cold meals, including both foods and drinks. Cold meals were defined as those served below room temperature, such as cold salads, cold sandwiches, sushi and cold milk. Similarly, we asked about the frequency of consuming entirely or mostly hot meals, also including foods and drinks. Hot meals were defined as those served at or above 30–40°C (86–104°F), such as warm sandwiches, rice dishes with cooked vegetables and warm soups. For both cold and hot meal questions, participants selected from the following multiple-choice options: Never or less than once per month, 1–3 times per month, once per week, 2–4 times per week, 5–6 times per week, 1–2 times per day, 3–5 times per day or 6+ times per day.

#### Assessment of ice cream consumption

Participants were given the same response options as described for cold drinks to indicate the frequency of their regular ice cream consumption during the summer and winter over the past 12 months.

### Assessment of outcomes proposed in the study

#### Assessment of depressive symptoms

Depressive symptoms were evaluated through self-reported questionnaires utilising the six-item short form of the Center for Epidemiologic Studies Depression Scale. The Center for Epidemiologic Studies Depression Scale is recognised as a valid and reliable tool for assessing depression symptoms^(23,24)^. The questionnaire included the six items to gauge depressive symptoms: (1) ‘you felt depressed’, (2) ‘your sleep was restless’, (3) ‘you enjoyed life’, (4) ‘you had crying spells’, (5) ‘you felt sad’ and (6) ‘you felt that people disliked you’. Responses were scored on a three-point Likert scale: 0 = ‘none of the time’, 1 = ‘some of the time’, 2 = ‘a moderate amount of time’ and 3 = ‘most of the time’. Participants who scored 5 or higher were considered to have elevated depressive symptoms^(24,25)^.

#### Assessment of anxiety

The Generalised Anxiety Disorder 7-item scale (GAD-7) is a brief self-report tool used to assess the severity of GAD. It consists of seven items based on Diagnostic and Statistical Manual of Mental Disorders-IV criteria and demonstrates strong internal consistency (*α* = 0·89–0·92) and criterion validity for identifying probable cases of GAD^(26,27)^. The total score ranges from 0 to 21, with a cut-off of 10 indicating possible GAD. In this study, participants scoring 10 or above were classified into the possible GAD group, while those scoring below 10 were classified as Non-GAD. This cut-off has a sensitivity of 89 % and specificity of 82 % for detecting possible GAD^(27)^.

#### Insomnia assessment

Insomnia was evaluated using the five-item Women’s Health Initiative Insomnia Rating Scale, a validated measure known for its high internal consistency^(28,29)^. The scale includes questions addressing difficulty falling asleep, waking up multiple times during the night, waking up too early, difficulty returning to sleep and overall sleep quality over the past four weeks. A score above nine on the Women’s Health Initiative Insomnia Rating Scale indicates clinically significant insomnia.

#### Assessment of abdominal fullness sensation and gas

We asked participants how often they experienced a sensation of abdominal fullness (feeling full) and gas in the past 12 months. They were given the options of ‘never,’ ‘rarely,’ ‘sometimes,’ ‘often’ and ‘always.’

#### Assessment of sensation of cold hands

To evaluate participants’ sensation of cold hands, we asked about the frequency with which they experienced this condition at indoor room temperature over the past 12 months. Responses were categorised as follows: Never, Rarely, Sometimes, Often or Always.

#### Assessment of covariates

Demographic characteristics and health status, including chronic conditions, were self-reported in the current survey and gathered from new participants. These characteristics included age, height, weight, place of birth, years of residence in the USA, primary language spoken at home, race and geographic location of residence. Physical activity levels were assessed using a validated questionnaire from the Multiethnic Cohort Study^(30,31)^. Smoking status and intensity were measured with a questionnaire from the Women’s Healthy Eating and Living Study, which has been shown to be associated with mortality risk^(32)^. Frequency of alcohol intakes and coffee drinking in the past 12 months were assessed in the survey. Living alone status was evaluated as a dichotomous variable (yes or no). Lastly, we assessed stressful life events using a twelve-item questionnaire adapted from the Alameda County Epidemiologic Study^(33)^, which was previously employed in Women’s Health Initiative (2014)^(34)^.

### Sample size and power calculations

Preliminary data showed correlations between cold/hot consumption and health outcomes ranging from 0·15 to 0·4, depending on race/ethnicity (White *v*. Asian). Based on a power calculation assuming a two-sided test at *α* = 0·05, a sample size of 193 provides 80 % power to detect a correlation of 0·2. More than 200 Asians and 200 Whites completed all relevant questionnaires. Because some correlations may be below 0·2, we also report marginal significance at the *α* = 0·10 level, which is sometimes used in studies with limited sample size^(35)^.

In addition to analysing cold/hot consumption as continuous variables, we also examined them as categorical variables. Power calculations were conducted using two-sample t-tests to compare mean health outcomes across tertiles of the cold/hot consumption score, focusing on two pairwise comparisons: tertile 3 *v*. 1 and tertile 2 *v*. 1. To account for multiple comparisons, we set the significance level at 0·025 (two-sided) and aimed for 80 % power. Under these assumptions, a sample size of twenty-one per group is sufficient to detect a mean difference of 1 when the sd is 1. If the sd is 1·5, the required sample size increases to forty-five per group. Based on our preliminary data, sd for mental health outcomes ranged from 0·6 to 1·2, while sd = for GI health outcomes ranged from 0·05 to 0·15.

Given these estimates, our sample size provides approximately 80 % power for the tertile analyses in both Asians and Whites, as well as for continuous analyses when the correlation is ≥ 0·2.

### Statistical analysis

To assess differences in baseline characteristics, we employed the χ^2^ test for categorical variables and *t* tests or ANOVA for normally distributed continuous variables and employed Wilcoxon rank sum tests or K-W tests for non-normally distributed continuous variables.

We created several derived consumption scores to reflect cold, hot and overall consumption. Based on the reported frequency of cold water and cold drink consumption, we created a ‘cold drink score’ by summing the frequencies for both. Similarly, a ‘hot drink score’ was calculated as the sum of the frequencies for hot water and hot drinks. To assess total cold consumption, we combined the cold drink score with the frequency score for cold meals. Similarly, total hot consumption was calculated by combining the hot drink score with the frequency score for hot meals. Finally, we created an overall cold–hot consumption score by combining both the cold and hot consumption scores, treating the cold score as positive and the hot score as negative.

Associations between cold exposures and various outcomes were examined separately in Asians and Whites. We used linear regression models, controlling for confounding factors. In model 1, we control for basic confounding factors including age, gender, BMI, physical activity, joint categories of smoking status and intensity and Asian subgroups (in Asian stratified analyses). In model 2, in addition to covariates included in model 1, we controlled for living-alone status, frequency of alcohol and coffee intake and number of lifetime stressful events. Because the results from model 1 and model 2 were not materially different in terms of beta estimates – though the *P* values were slightly attenuated for some estimates in model 2 – we chose to use model 1 for all linear regression analyses to conserve statistical power. The selection of covariates was guided by a priori hypotheses informed by existing literature on similar outcome variables.

As described, we created several exposure variables related to cold and hot consumption and examined them separately in multivariable models. We manually adjusted these exposure variables as follows: cold drink and hot drink scores from the same season were adjusted simultaneously; cold meal and hot meal frequency scores from the same season were adjusted simultaneously and total cold and hot consumption scores from the same season were adjusted simultaneously. Lastly, ice cream consumption and the total cold and hot composite scores from each season were analysed separately. Because the results related to ice cream were no longer significant after using model 2 mentioned above, they were ultimately excluded from the analyses.

We further conducted stratified analyses. We first stratified the analysis based on participants’ cold hand status and further by Asian subgroups (Chinese, Asian Indian, South Asian and other Asian groups for exploratory analyses). Forest plots were presented to show some subgroup associations. To evaluate statistical significance across different strata and test for interaction between subgroups, we used the *P* value for the interaction term in a model that also included the main effects.

## Results

### Participant characteristics

As shown in Table 1, when comparing the 212 Asian Americans to the 203 White Americans, the median age, BMI and physical activity levels were all higher among Whites. Additionally, Asians had a higher proportion of females, individuals born outside the USA (46 %) and migrants (39 %). Migrants were defined as first-generation immigrants born outside of the USA who arrived in the USA after the age of 12, since early childhood plays an important role in shaping adult lifestyle habits^(36,37)^.

**Table 1.** General characteristics among Asians and Whites enrolled in the Healthy Ageing Study (Median values and interquartile ranges; numbers and percentages)

	Total Asian		Total White		
	*n* 212		*n* 203		
	Median	IQR	Median	IQR	*P*_for racial comparison_
Age (median, inter-quartile range)^*^	31	25–41	50	33–60	**<** 0·0001
BMI (median, inter-quartile range)	22	21–26	25	22–29	**<** 0·0001
Physical activity (met-h/week)^‡^	4·4	2·4–8·0	5·8	2·9–9·4	0·005
	*n*	%	*n*	%	
Birth sex^§^					
Female	171	81	140	69	0·006
Male	41	19	63	31	
Born outside of the USA (*n*, %)					
No	114	54	197	97	**<** 0·0001
Yes	98	46	6	3	
Migrant (*n*, %)^ǁ^					
No	128	61	198	98	**<** 0·0001
Yes	82	39	5	2	
Smoking status (*n*, %)					
Never smoker	196	92	150	74	**<** 0·0001
Past smoker	14	7	39	19	
Current smoker	2	1	14	7	
US states (*n*, %)					
California	76	37	100	49	0·02
Southern	42	21	41	20	
Midwestern	26	13	17	8	
Northeastern	46	23	24	12	

IQR, interquartile range.

* Continuous variables are presented as median (inter-quartile range).

‡ Met-hour for moderate and vigorous physical activity/week.

§ Categorical variables are presented as *n* (%).

ǁ The sum of the numbers in the rows for migrants does not equal the total due to missing values.

Compared with Whites, Asians also had fewer past and current smokers, and fewer participants from California, but more from Northern states.

Table 2 shows the overall patterns and disparities in cold and hot consumption between Asians and Whites. In general, compared with Whites, Asians had lower intakes of cold drinks and cold meals, as well as a lower percentage of individuals reporting high consumption of these items. In contrast, Asians had higher intake of hot meals and a greater percentage of individuals reporting high hot meal consumption in both winter and summer. Hot drink intake was also higher among Asians in summer, but not in winter.

**Table 2.** Cold and hot drinks and meal score in Asians and Whites (Median values and interquartile ranges; numbers and percentages)

	Total Asian (*n* 212)		Total White (*n* 203)		*P*_for racial comparison_
	*n*/Median	%/IQR	*n*/Median	%/IQR	
Cold drink score^*^ in winter, median (IQR)	7	3–10	9	6–12	< 0·0001^†^
Cold drink >= 5–6 times/week in winter, *n* (%)	63	30 %	90	44 %	0·009
Cold meal score in winter, median (IQR)	2	1–3	3	2–4	**<** 0·0001
Cold meal >= 3 times/week in winter, *n* (%)	43	20 %	84	41 %	**<** 0·0001
Hot drink score^‡^ in winter, median (IQR)	7	5–10	7	6–8	0·5
Hot drink >= 5–6 times/week in winter, *n* (%)	58	27 %	37	18 %	0·5
Hot meal score in winter, median (IQR)	6	5–6	5	4–6	**<** 0·0001
Hot meal >= 1–2 times/d in winter, *n* (%)	140	66 %	100	49 %	0·009
Cold drink score in summer, median (IQR)	9	6·5–12	11	8–13	**<** 0·0001
Cold drink >= 5–6 times/week in summer, *n* (%)	98	46 %	134	66 %	0·02
Cold meal score in summer, median (IQR)	3	2–4	4	3–6	**<** 0·0001
Cold meal >= 3 times/week in summer, *n* (%)	93	44 %	146	72 %	**<** 0·0001
Hot drink score in summer, median (IQR)	5	3–7	7	5–7	0·06
Hot drink >= 5–6 times/week in summer, *n* (%)	22	10 %	12	6 %	0·002
Hot meal score in summer, median (IQR)	5	4–6	4	3–6	**<** 0·0001
Hot meal >= 1–2 times/d in summer, *n* (%)	95	45 %	51	25	**<** 0·0001
Total cold consumption score^§^ (drinks plus meals) in winter, median (IQR)	6·7	4·2–9·4	8·6	6·5–11·0	**<** 0·0001
Total hot consumption score^ǁ^ (drinks plus meals) in winter, median (IQR)	11·0	9·8–13·4	10·6	8·7–11·7	0·004
Total cold consumption score (drinks plus meals) in summer, median (IQR)	9·4	7·0–11·6	11·5	9·4–13·7	**<** 0·0001
Total hot consumption score (drinks plus meals) in summer, median (IQR)	9·2	6·9–11	8·6	6·6–9·9	0·02
Composite score^¶^ (total cold plus hot consumption scores) in winter, median (IQR)	–4·2	–8·1 to –0·6	–1·25	–4·9–1·7	**<** 0·0001
Composite score (total cold plus hot consumption scores) in summer, median (IQR)	0·4	–2·5 to 3·2	3·1	0·14–5·6	**<** 0·0001
Ice cream intake >= 3 times/week in winter (*n*, %)	26	12 %	30	15 %	0·5
Ice cream intake >= 3 times/week in summer (*n*, %)	41	19 %	54	27 %	0·06

IQR, interquartile range.

* Cold drink score summarises the combined consumption of cold drinks and cold water.

‡ Hot drink score summarises the combined consumption of hot drinks and hot water.

§ The total cold consumption score summarises cold drink score and cold meal frequency score.

ǁ The total hot consumption score summarises hot drink score and hot meal frequency score.

¶ The composite score combines total cold and hot consumption scores, with the cold score treated as positive and the hot score as negative.

Furthermore, similar patterns were observed for total cold and hot consumption, as well as for the composite score (total cold score minus total hot score). Asians had lower total cold consumption, higher hot consumption and therefore lower composite scores compared with Whites.

Table 3 illustrates the distribution of the proposed outcomes across Asians and Whites. Overall, there were no significant differences in depression scores or the proportion of individuals with depressive symptoms, anxiety scores or the prevalence of anxiety disorders, insomnia symptoms or reports of experiencing gas. Only insomnia scores and the proportion of individuals often or always reporting abdominal fullness were slightly higher among Whites compared with Asians.

**Table 3.** Ageing-related subclinical health conditions among Asians and Whites in the whole Healthy Ageing Study (Mean values and standard deviations; numbers and percentages)

	Total Asian (*n* 212)		Total White (*n* 203)		*P*_for comparison_
	*n*/Mean	%/sd	*n*/Mean	%/sd	
Mental health					
Depression score (mean, sd)	3·8	3·6	4·1	3·7	0·4
Classified with depression symptoms (score >= 5), *n* (%)	67	32 %	78	38 %	0·15
Anxiety score (mean, sd)	4·6	4·9	4·5	4·7	0·8
Classified with anxiety disorder (score >= 8), *n* (%)	48	23 %	42	21 %	0·62
Insomnia score (mean, sd)	7·4	3·3	8·1	3·7	0·07
Classified with insomnia symptoms (score >= 9), *n* (%)	69	33 %	78	38 %	0·21
Gut health					
Often or always with sensation of abdominal fullness, *n* (%)	65	31 %	76	37 %	0·09
Often or always experiencing gas, *n* (%)	19	9 %	18	9 %	0·7
Experience cold hands (sometimes, often or always), *n* (%)	145	68 %	132	65 %	0·29

### Multivariable-adjusted analyses

#### Cold consumption and the combination of cold and hot consumption among Asians (Table 4)

**Table 4.** Cold consumption and the combination of cold and hot consumption in relation to multiple health outcomes among Asians (All associations between exposures and outcomes are presented as beta estimates (95 % confidence interval; *P* value))

	Cold drink frequency score^*^							Total cold consumption^†^ score excluding ice cream			Composite score^‡^ (total cold minus total hot)		
		Winter			Summer			Summer			Summer		
Outcome variables	Consumption variable format	*β*	95% CI	*P* value	*β*	95% CI	*P* value	*β*	95% CI	*P* value	*β*	95% CI	*P* value
Mental health													
Depression score	Tertile 1	Ref			Ref			Ref			Ref		
	Tertile 2	–0·15	–1·29, 0·99	0·79	0·97	–0·25, 2·18	0·56	0·27	–0·83, 1·37	0·63	0·17	–1·03, 1·37	0·78
	Tertile 3	0·37	–0·94, 1·66	0·57	0·35	–0·83, 1·53	0·12	0·58	–0·78, 1·94	0·39	–0·57	–1·97, 0·82	0·38
	Continuous	0·05	–0·07, 0·17	0·41	0·07	–0·07, 0·21	0·30	0·07	–0·08, 0·22	0·30	–0·001	–0·13, 0·12	0·91
Anxiety score	Tertile 1	Ref			Ref			Ref			Ref		
	Tertile 2	0·47	–1·03, 1·97	0·54	2·68	1·12, 4·24^‡^	0·0008	1·53	0·11, 2·95	0·03	1·08	–0·48, 2·64	0·17
	Tertile 3	0·94	–0·75, 2·62	0·27	1·29	–0·22, 2·82	0·09	1·63	–0·12, 3·39	0·06	–0·91	–2·72, 0·89	0·32
	Continuous	0·13	–0·04, 0·30	0·13	0·20	0·02, 0·38	0·03	0·24	0·05, 0·44	0·01	0·005	–0·16, 0·17	0·95
Insomia score	Tertile 1	Ref			Ref			Ref			Ref		
	Tertile 2	0·88	–0·17, 1·92	0·10	1·36	0·25, 2·45	0·02	0·48	–0·53, 1·49	0·35	–0·17	–1·28, 0·93	0·76
	Tertile 3	0·86	–0·32, 2·04	0·15	1·26	0·19, 2·33	0·02	1·22	–0·03, 2·47	0·06	0·27	–1·01, 1·56	0·68
	Continuous	0·12	0·00, 0·23	0·05	0·14	0·02, 0·26	0·02	0·17	0·03, 0·31	0·02	0·05	–0·06, 0·16	0·36
Gut health													
Gas	Tertile 1	Ref			Ref			Ref			Ref		
	Tertile 2	–0·25	–0·54, 0·03	0·08	–0·01	–0·32, 0·29	0·93	–0·08	–0·29, 0·27	0·95	0·13	–0·17, 0·43	0·39
	Tertile 3	–0·30	–0·61, 0·02	0·06	0·31	0·02, 0·60	0·04	0·35	0·01, 0·69	0·04	0·05	–0·34, 0·36	0·98
	Continuous	–0·02	–0·06, 0·008	0·13	0·01	–0·03, 0·05	0·6	0·02	–0·02, 0·06	0·25	0·01	–0·02, 0·04	0·49
Sensation of abdomnial fullness	Tertile 1	Ref			Ref			Ref			Ref		
	Tertile 2	0·14	–0·16, 0·45	0·37	0·28	–0·05, 0·61	0·09	0·20	–0·10, 0·50	0·19	0·47	0·15, 0·79	0·004
	Tertile 3	0·19	–0·15, 0·54	0·27	0·45	0·11, 0·75	0·009	0·18	–0·18, 0·56	0·31	0·38	0·01, 0·75	0·04
	Continuous	0·03	–0·06, 0·01	0·14	0·05	0·01, 0·86	0·02	0·04	0·00, 0·08	0·04	0·03	–0·005, 0·06	0·09

* The cold drink frequency score summarises the combined consumption of cold drinks and cold water.

† The total cold consumption score summarises cold drink score and cold meal frequency score.

‡ The composite score combines total cold and hot consumption scores, with the cold score treated as positive and the hot score as negative.

Multivariable included age, BMI, physical activities, smoking status and Asian subgroups. In the multivariable models, total cold and hot drink frequency scores during the same season were adjusted simultaneously in the model; total cold and hot consumption scores during the same season were also adjusted simultaneously and total composite scores for summer and winter were adjusted simultaneously in the model.

Because no significant associations were observed between hot consumption and the proposed outcomes among Asians, those results are not shown. The analyses focus on cold consumption variables and the composite score. Since most significant findings for total cold consumption and the composite score occurred in summer, winter results are not presented. Cold or hot meal consumption showed few significant associations with the outcomes and is therefore not presented.

For mental health outcomes, particularly depression, there were no consistent trends with any cold consumption scores or the composite score. In contrast, stronger and more consistent associations were observed for anxiety, particularly in relation to cold drink intake and total cold consumption during summer. The results were consistent whether these cold consumption scores were analysed as tertiles or as continuous variables. Compared with tertile 1, tertiles 2 and 3 were associated with a 1·29 to 2·68-point increase in anxiety scores. Increased cold meal consumption in summer was marginally associated with anxiety score when analysed as a continuous variable (data not shown).

For insomnia, both cold drink consumption and total cold consumption were associated with increased insomnia scores when analysed as tertiles or as continuous variables. For example, in summer, compared with tertile 1, cold drink consumption was associated with a 1·36-point increase in insomnia score for tertile 2 (95 % CI: 0·25, 2·45) and a 1·26-point increase for tertile 3 (95 % CI: 0·19, 2·33). Cold meal consumption in both winter and summer was significantly associated with insomnia only when it was analysed as a continuous variable (data not shown).

For gut health, cold drinks in summer, total cold consumption in summer and the composite score in summer were mostly associated with the sensation of abdominal fullness, whether analysed as tertiles or as a continuous variable. Associations with gas were less consistent, with significant findings only observed in tertile 3 compared with tertile 1 for cold drinks and total cold consumption in summer. An opposite association was observed for cold drinks in winter, where higher consumption was linked to reduced gas.

#### Hot consumption and the combination of cold and hot consumption among Whites (Table 5)

**Table 5.** Hot consumption and the combination of cold and hot consumption in relation to multiple health outcomes among Whites (All associations between exposures and outcomes are presented as beta estimates (95 % confidence interval; *P* value))

Hot drink frequency score^*^							Total hot consumption^†^ score			Composite score^‡^ (total cold minus total hot)			
Outcome variables	Consumption variable format	Winter			Summer			Winter			Winter		
		*β*	*95%CI*	p value	*β*	*95%CI*	p value	*β*	*95%CI*	p value	*β*	95%CI	P value
Mental health													
Depression score	Tertile 1	Ref			Ref			Ref			Ref		
	Tertile 2	–1·20	–2·44, −0·09^§^	0·06	–1·36	–2·63, −0·09	0·04	–0·79	–1·96, 0·37	0·18	1·88	0·46, 3·31	0·009
	Tertile 3	–1·73	–3·28, −0·18	0·02	–1·96	–3·55, −0·38	0·01	–1·20	–2·67, 0·27	0·10	1·84	0·48, 3·21	0·009
	Continuous	–0·16	–0·35, 0·03	0·09	–0·34	–0·56, −0·11	0·003	–0·11	–0·28, 0·06	0·22	0·11	0·00, 0·22	0·05
Anxiety score	Tertile 1	Ref			Ref			Ref			Ref		
	Tertile 2	–0·81	–2·30, 0·68	0·28	–1·23	–2·66, 0·41	0·15	–0·64	–2·03, 0·76	0·37	2·12	0·43, 3·81	0·01
	Tertile 3	–1·95	–3·06, 0·67	0·21	–1·46	–3·37, 0·46	0·14	–1·69	–3·44, 0·07	0·06	2·34	0·71, 3·97	0·005
		–0·10	–0·33, 0·12	0·36	–0·26	–0·53, 0·00	0·05	–0·08	–0·28, 0·13	0·46	0·13	0·00, 0·26	0·05
Insomnia score	Tertile 1	Ref			Ref			Ref			Ref		
	Tertile 2	0·39	–0·86, 1·65	0·50	–0·81	–2·14, 0·52	0·23	0·32	–0·86, 1·51	0·59	2·10	0·65, 3·55	0·004
	Tertile 3	–1·87	–3·44, −0·29	0·02	–0·88	–2·54, 0·77	0·29	–1·32	–2·81, 0·18	0·08	1·88	0·49, 3·27	0·008
	Continuous	–0·23	–0·42, −0·04	0·02	–0·19	–0·42, 0·04	0·10	–0·15	–0·32, 0·02	0·09	0·09	–0·02, 0·21	0·11
Gut health													
Gas	Tertile 1	Ref						Ref			Ref		
	Tertile 2	0·01	–0·27, 0·30	0·92	–0·12	–0·42, 0·18	0·42	–0·02	–0·29, 0·25	0·88	0·08	–0·24, 0·41	0·61
	Tertile 3	–0·52	–0·90, −0·18	0·003	–0·14	–0·52, 0·23	0·45	–0·44	–0·77, −0·11	0·009	0·31	0·008, 0·62	0·05
	Continuous	–0·05	–0·09, −0·01	0·02	–0·02	–0·07, 0·04	0·51	–0·04	–0·08, 0·00	0·02	0·03	0·00, 0·05	0·005
Sensation of abdominal fullness	Tertile 1	Ref			Ref			Ref			Ref		
	Tertile 2	–0·08	–0·38, 0·21	0·57	–0·22	–0·52, 0·08	0·15	0·11	–0·17, 0·39	0·45	–0·02	–0·36, 0·31	0·89
	Tertile 3	–0·05	–0·40, 0·30	0·76	–0·31	–0·69, 0·07	0·11	–0·01	–0·35, 0·32	0·94	0·04	–0·37, 0·27	0·78
	Continuous	–0·00	–0·04, 0·04	0·95	–0·05	–0·10, 0·01	0·10	–0·001	–0·04, 0·04	0·94	0·01	–0·04, 0·02	0·39

* The hot drink frequency score summarises the combined consumption of hot drinks and hot water.

† The total hot consumption score summarises hot drink score and hot meal frequency score.

‡ The composite score combines total cold and hot consumption scores, with the cold score treated as positive and the hot score as negative.

Multivariable included age, BMI, physical activities, smoking status and Asian subgroups. In the multivariable models, total cold and hot drink frequency scores during the same season were adjusted simultaneously in the model; total cold and hot consumption scores during the same season were also adjusted simultaneously and total composite scores for summer and winter were adjusted simultaneously in the model.

Because we did not observe significant trends between cold drinks or total cold consumption and most of the proposed outcomes, these results are not presented but were included as covariates. Additionally, since most significant findings for total hot consumption and the composite score were observed in winter, summer results are not shown. Most outcomes associated with hot or cold meals were not statistically significant and, therefore, are not presented.

Hot consumption, particularly hot drinks, was associated with reduced depression severity among White participants, while higher cold and lower hot consumption (as reflected in the composite score) was linked to increased depression. The associations between hot drinks and depression were consistent across both winter and summer, with stronger effects observed in summer. For example, those in the highest tertile of summer hot drink intake had a 1·96-point lower depression score compared with the lowest tertile (95 % CI: −3·55, −0·38). Total hot consumption showed weaker associations. The composite score suggests that higher cold consumption and lower hot consumption were associated with increased depression scores, whether analysed as categorical variables (tertiles) or as continuous variables.

For anxiety scores, only the composite score showed consistent results when analysed both as tertiles and as a continuous variable. For other scores, significant associations were observed only in specific cases – such as hot drink consumption analysed as a continuous variable in summer or total hot consumption at tertile 3 in winter compared with tertile 1.

For insomnia, results were consistent across different consumption scores, showing that higher hot consumption was associated with reduced insomnia – whether analysed as tertile variables or as a continuous variable, though not always both.

For gut health, most of the significant results were observed in the associations between gas and various consumption scores during winter. All of these findings consistently showed that higher hot consumption was associated with reduced severity of gas. The strongest effect was seen in tertile 3 of hot drink consumption in winter (*β* = –0·52; 95 % CI: –0·90, –0·18), compared with tertile 1. For abdominal fullness, a marginally significant inverse association was observed only for hot drinks in summer and only when analysed as a continuous variable.

#### Modification by cold hand status

**Figure 1.**
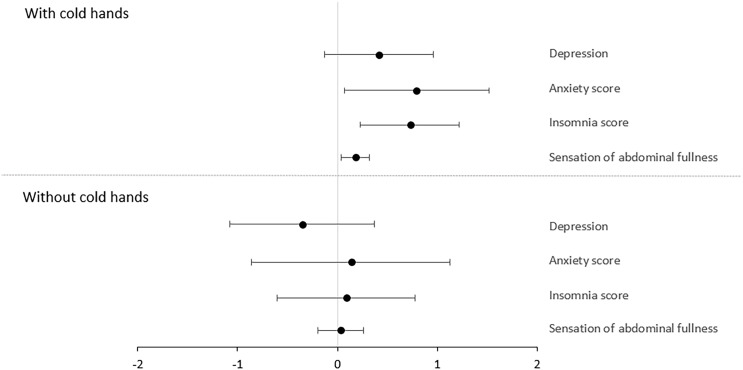
Total cold consumption in summer in relation to multiple health outcomes in Asians. Covariates in the multiviable model included age, BMI, physical activities, smoking status and Asian subgroups.

**Figure 2.**
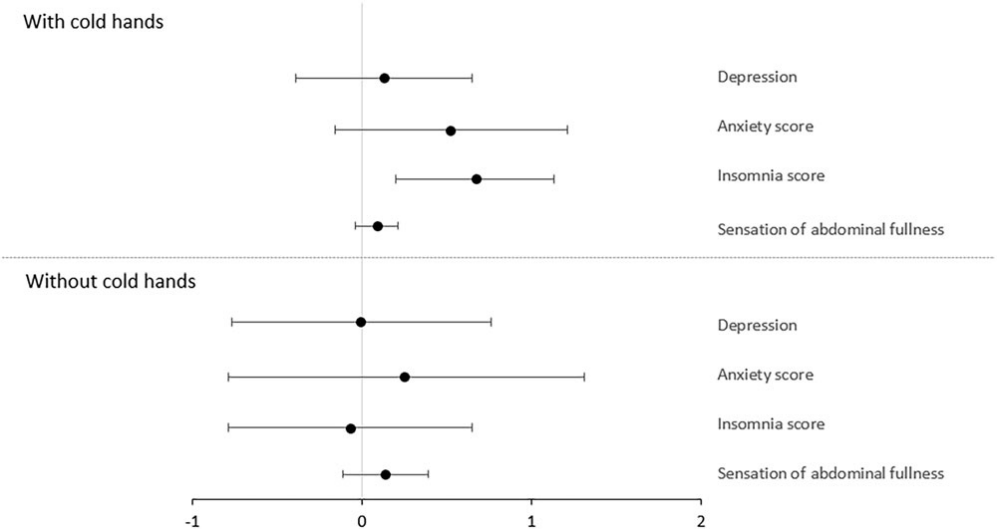
Total cold consumption in winter in relation to multiple health outcomes in Asians. Covariates in the multiviable model included age, BMI, physical activities, smoking status and Asian subgroups.

Figures 1 and 2 examined the effect modification by cold hand status for Asians. Figure 1 illustrates the cold consumption score in summer with different outcomes. As shown in Figure 1, the total cold consumption score showed a trend towards significance with all proposed outcomes in the cold hand group but not in the non-cold hand group. The *P* value for interaction was < 0·05 for most of the outcomes. Figure 2 displays the cold consumption score in winter across different outcomes, showing similar trends to Figure 1 but with less significance and smaller magnitudes for several outcomes.

In addition, we examined whether cold hand status modified the associations among Whites. As shown in Table 6, among those who reported sometimes, often or always experiencing cold hands, the composite score at tertiles 2 and 3 (compared with tertile 1) was significantly associated with higher depression, anxiety and insomnia scores, though not with gas or abdominal fullness. These associations were not observed among individuals who never or rarely experienced cold hands. While the tertile analysis suggested a trend towards effect modification, the associations were not significant when the composite score was analysed as a continuous variable, indicating that the modification effect may be limited to non-linear relationships.

**Table 6.** Combined cold and hot consumption (composite score)^*^ during winter in relation to multiple health outcomes among Whites, stratified by cold hand status (All associations between exposures and outcomes are presented as beta estimates (95 %confidence interval; *P* value))

		Without coldhands (*n* 71) (never, rarely)			With coldhands (*n* 132) (sometimes, often, always)				
Outcome variables	Consumption variable format	*β*	95 % CI	*P* value	*β*	95 % CI	*P* value		
Mental health									
Depression score	Tertile 1	Ref			Ref				
	Tertile 2	0·93	–1·45, 3·31	0·44	2·21	0·35, 4·07	0·02		
	Tertile 3	0·82	–1·69, 3·33	0·51	2·17	0·39, 3·98	0·02		
	Continuous	0·13	–0·08, 0·34	0·21	0·09	–0·06, 0·24	0·21		
Anxiety score	Tertile 1	Ref			Ref				
	Tertile 2	0·68	–1·97, 3·35	0·61	2·70	0·46, 4·93	0·02		
	Tertile 3	1·19	–1·61, 3·99	0·64	2·94	0·78, 5·09	0·01		
	Continuous	0·13	–0·10, 0·37	0·26	0·13	–0·04, 0·32	0·13		
Insomnia score	Tertile 1	Ref			Ref				
	Tertile 2	0·11	–2·37, 2·58	0·93	2·96	1·17, 4·76	0·001		
	Tertile 3	0·61	–2·01, 3·22	0·64	2·31	0·58, 4·04	0·009		
	Continuous	0·09	–0·13, 0·31	0·43	0·09	–0·06, 0·24	0·23		
Gut health									
Gas	Tertile 1	Ref			Ref				
	Tertile 2	–0·11	–0·62, 0·40	0·65	0·19	–0·25, 0·64	0·39		
	Tertile 3	0·31	–0·22, 0·85	0·25	0·31	–0·11, 0·72	0·15		
	Continuous	0·02	–0·02, 0·07	0·32	0·03	–0·00, 0·06	0·12	0·7	0·9
Sensation of abdominal fullness	Tertile 1	Ref			Ref				
	Tertile 2	–0·20	–0·79, 0·39	0·51	0·17	–0·22, 0·56	0·40		
	Tertile 3	–0·21	–0·84, 0·41	0·50	0·07	–0·30, 0·43	0·73		
	Continuous	–0·02	–0·07, 0·04	0·50	0·004	–0·03, 0·04	0·82		

Multivariable included age, BMI, physical activities and smoking status. In the multivariable model, total composite scores for summer and winter were adjusted simultaneously in the model.

* The composite score combines total cold and hot consumption scores, with the cold score treated as positive and the hot score as negative.

### Additional analyses among Asian subgroups

We conducted additional exploratory analyses among Asian subgroups. As shown in online Supplementary Table 1, among Asians, the majority are Chinese (77, 36 %), followed by Asian Indians (43, 20 %), South Asians (55, 26 %) and other Asians (37, 17 %). Chinese participants had the lowest percentages of individuals consuming high levels of cold drinks and cold meals. They also reported the lowest depression and anxiety scores, as well as the lowest prevalence of abdominal fullness (online Supplementary Table 2). Furthermore, when stratifying by Asian subgroups, significant associations between cold consumption and adverse outcomes were observed only among Asian Indians (online Supplementary Figure 1).

## Discussion

In this study of 212 Asian and 203 White American participants, we examined the associations between cold consumption (including cold drinks/water and cold meals) and hot consumption (including hot drinks such as coffee and tea, as well as hot meals) and a range of health outcomes. These outcomes included mental health measures (depression, anxiety and insomnia) and gut health indicators (abdominal fullness and gas), analysed separately for Asian and White participants. We observed distinct patterns between cold and hot consumption and health outcomes across the two racial/ethnic groups. Among Asians, cold – but not hot – consumption was independently associated with multiple outcomes, including anxiety, insomnia and abdominal fullness, but not depression. Both linear and non-linear analyses supported these associations, particularly in summer. Additionally, the impact of cold consumption on gut health was primarily reflected in increased abdominal fullness during the summer. In contrast, among Whites, hot – but not cold – consumption was independently and inversely associated with several health outcomes, including depression, anxiety, insomnia and gas. The strongest association was observed for depression, followed by insomnia and gas, with consistent findings across both linear and non-linear analyses, as well as across seasons. Unlike in Asians, the impact on gut health among Whites was mainly reflected in gas, not abdominal fullness. The composite score (reflecting higher cold and lower hot consumption as the score increases) was associated with all proposed outcomes except abdominal fullness, suggesting that the combined effect of cold and hot intake remained important even when individual cold variables were not independently significant. The associations for the composite score were mainly significant in winter, but not in summer, among Whites.

Additionally, ‘cold hands’ modified these associations in both Asians and Whites, with stronger links to nearly all adverse outcomes observed among individuals with cold hands, but no significant associations among those without this trait. Overall, findings from both Asian and White participants support our hypothesis that cold consumption is more harmful, whereas hot consumption is more beneficial. However, sensitivity to cold, hot or combined effects may vary by racial groups.

Depression, anxiety, insomnia and gut health are critical areas of study due to their close interrelationships and strong associations with both ageing-related morbidity and mortality, as well as with substance use disorders and suicide^(38–47)^. Thus, identifying novel dietary habits that are strongly associated with these conditions has profound public health impacts.

Numerous studies have shown that outdoor environmental cold exposure can lead to vascular diseases, pain and an increased incidence of fractures^(48–52)^. Other human studies have found cold water consumption was associated with lower core body temperature and higher blood pressure in the elderly^(11)^ and increased digestive discomfort, such as bloating and cramping, in individuals with irritable bowel syndrome^(53)^. However, to our knowledge, this is the first study to investigate the relationship between cold and hot consumption and multiple health outcomes within a single study of the general Asian and White American population, covering young adults, middle-aged adults and young elderly adults aged 18–65 years. Our group previously demonstrated that cold drinks/water consumption was associated with dysmenorrhoea in both Whites and Asians^(18)^. In the current study, we extended our investigation to include not only cold drinks but also the combination of cold meals and drinks, the combined effects of cold and hot consumption and additional health outcomes.

There are many experimental studies that help explain why cold consumption may lead to multiple adverse health outcomes in Asians and why hot consumption may help reduce these adverse outcomes in Whites, as investigated in the current study. The mechanisms underlying cold consumption primarily involve, but are not limited to, disturbances in the gut–brain axis, reduced systemic blood flow, destabilisation of the hypothalamic–pituitary–adrenal axis and imbalances in neurotransmitter levels. In contrast, the benefits associated with hot consumption may stem from the opposite effects of these mechanisms.

Prior research has shown that the gut–brain axis is a key area of study^(54)^ in understanding how cold consumption may contribute to both digestive discomfort – such as abdominal fullness and gas – and mental health symptoms observed in this study. For instance, hand immersion in ice water has been shown to raise gut permeability^(55)^, and cold water intake can worsen GI symptoms in individuals with irritable bowel syndrome^(53)^. Animal studies further show that cold water increases colon transit time^(56,57)^, disrupts the microbiome^(9,10)^ and promotes inflammation^(9)^, which can destabilise the hypothalamic–pituitary–adrenal axis^(58,59)^. Hypothalamic–pituitary–adrenal axis is a key stress-response system, and its destabilisation can further disturb neurotransmitter balance synthesis^(60,61)^ and contributing to symptoms of depression, anxiety and sleep disturbances^(62–66)^.

Cold stress – whether from cold beverages, environmental exposure or immersion – can negatively impact mental health by disrupting several physiological systems in addition to GI system. It reduces blood flow and microcirculation, leading to vasoconstriction^(10,11,49)^, which limits oxygen and nutrient delivery to the brain^(67,68)^. This can impair neurotransmitter production, including serotonin and GABA^(69,70)^, both crucial for mood regulation and sleep^(65,71–73)^. Reduced oxygenation and elevated blood pressure from vasoconstriction are also linked to insomnia^(74,75)^. Additionally, cold intake also lowered core and GI temperatures^(11,76)^, activating the sympathetic nervous system^(11,77)^ and increasing arousal-related hormones like cortisol and norepinephrine^(78,79)^, which interfere with sleep quality^(80)^. These interconnected mechanisms help explain how cold consumption may contribute to depression, anxiety and insomnia.

Understanding the adverse effects of cold consumption helps clarify the potential benefits of warm or hot consumption, which often produces opposite physiological responses. Heat-based interventions – such as drinking warm or hot water, thermal therapy and warm water irrigation during surgery – have been shown to improve blood circulation, support GI function and help maintain core body temperature^(15–17)^. Enhanced circulation promotes oxygen and nutrient delivery, aiding tissue repair and reducing physiological stress^(68,81)^. Clinically, warm water irrigation during abdominal or pelvic surgeries helps prevent hypothermia and stabilise core temperature^(17,82)^. Additionally, warm water intake has been linked to improved gastric motility and reduced symptoms like bloating and cramping, especially in individuals with functional GI disorders^(16,83)^. These effects may also enhance mood and relaxation by activating the parasympathetic nervous system and lowering systemic stress levels^(84)^.

The differing associations between cold and hot consumption and adverse outcomes in Asians and Whites may stem from both physiological and cultural factors. Asians are more prone to diabetes than Whites, even at similar or lower BMI levels^(85,86)^, partly due to higher visceral fat and lower muscle mass^(87,88)^, which may also reduce thermogenic capacity^(89,90)^. Culturally, Whites tend to consume more cold items, while Asians prefer hot items, as shown in our study. Seasonal patterns also differ: cold consumption is more strongly linked to adverse outcomes in summer among Asians, while hot consumption shows stronger associations in winter among Whites. Although both groups consume more cold drinks in summer, the negative effects are more pronounced in Asians, likely due to greater sensitivity to cold exposure. While cold drinks can reduce hyperthermia risk^(91,92)^, our data suggest that excessive intake – especially among those with cold extremities – may be harmful. Whites may be more resilient to cold, which could explain the lack of summer impact but noticeable effects in winter, when cold stress and the demand for thermogenesis increase. This may also explain the benefits of hot consumption in winter, as it may alleviate cold stress. Nevertheless, despite greater physiological resistance, our composite score shows that high cold consumption combined with low hot consumption is still associated with adverse health outcomes among White participants.

Asians are not a homogeneous group. Among them, Chinese individuals reported the lowest cold consumption, which may partly explain their fewer adverse health outcomes (online Supplementary Tables 1 and 2). We found no significant associations between cold consumption and outcomes among Chinese, but did observe such associations in Asian Indians (online Supplementary Figure 1). These differences may be due to small subgroup sample sizes, varying cultural practices and influences from traditional Chinese medicine. Reverse causation is also possible – individuals, particularly Chinese, who are aware of the negative effects of cold consumption may reduce intake after experiencing symptoms. Early life cultural influence is evident, as we found that migrant status was inversely correlated with cold consumption among Asians (*r* = –0·2 to –0·5, *P* < 0·0001). Further research with larger, more diverse Asian samples is needed.

We observed significant or marginal effect modification by cold hand status. Stronger associations between cold consumption and the proposed outcomes were found among individuals who are more likely to have cold hand status. Cold hand status reflects disturbed peripheral blood circulation, as seen in Raynaud’s phenomenon^(93–95)^. In addition to the mechanisms mentioned above related to reduced blood flow, several human studies also support our findings. Individuals with existing poor blood circulation and ischaemia are more susceptible to depression, insomnia, anxiety and ischaemic pain^(96–98)^. Raynaud’s phenomenon, an extreme case of cold hands, has been extensively studied; however, less attention has been given to individuals who experience occasional or frequent cold hands without reaching the severity levels seen in Raynaud’s. If our study can be further confirmed, it will have wider clinical applications, as we can use cold hand status to predict individuals who are more susceptible to cold consumption.

Our study has several notable strengths. It is the first to comprehensively examine the relationship between cold and hot consumption and a wide range of important health outcomes among Asians and Whites living in the USA, a country with a high prevalence of cold drink and cold meal consumption. We documented disparities in cold and hot consumption and associated health outcomes among Asians and Whites. We offer insights that have been largely overlooked in the current literature, providing a critical foundation for future research in this area. Another significant strength of our study is its consideration of cold hand status, a potential early indicator of peripheral circulation issues. This is particularly valuable because cold hand status is not typically explored in Western medical studies, yet it has deep roots in traditional Chinese and Korean medicine and has been shown to be a strong predictor of various health conditions^(99–101)^. Our ability to link cold hand status as a predictor of cold consumption-related health outcomes adds an innovative dimension to the field, broadening the understanding of the physiological implications of cold exposure.

However, our study has certain limitations. As a cross-sectional study, it cannot establish causality, and future longitudinal studies with repeated measurements are needed to validate our findings. Additionally, all outcome measures, as well as cold and hot exposure and cold hand status, were self-reported rather than objectively measured. Inherent measurement error (such as recall bias and reporting inaccuracies) in nutritional questionnaire data collection is a limitation of this study. Such error can introduce unpredictable bias, potentially attenuating or inflating observed associations in unknown ways. Future longitudinal studies, studies with objective validation and intervention studies are warranted. Despite this, these self-reported outcomes have been widely used to predict ageing-related diseases and reflect subclinical conditions linked to serious health outcomes^(32,102–105)^. Cold and hot drink consumption^(6–8)^ and cold hand status^(99–101)^ are also recognised in traditional Chinese, Ayurvedic and Korean medicine for their predictive value. One might question whether we should adjust for total energy intake. However, only a small number of participants completed two 24-h recalls. Among them, cold consumption showed low correlation with total energy or macronutrient intake (*r* = 0·17, *P* = 0·12), and no correlation was found for hot drinks or meals. While some confounding is possible, it is likely minimal. Moreover, the contents of cold and hot drinks were not assessed. Whether those contents can confound the results will need to be evaluated in the future. Larger longitudinal studies with more comprehensive adjustments of covariates are recommended for future research. Lastly, in future studies with larger sample sizes, alternative approaches such as spline regression may allow for a more comprehensive and flexible assessment of potential non-linear relationships between cold/hot consumption and the health outcomes of interest.

In summary, our research is timely and relevant in the context of a growing body of evidence connecting environmental and lifestyle factors to health disparities. Recent trends in the USA show an alarming rise in the prevalence of poor mental and physical health, as well as other chronic conditions^(106–108)^, contributing to increased healthcare burdens and diminished quality of life. By addressing cold consumption, a largely modifiable behaviour, our findings have practical implications for public health interventions targeting specific cultural and ethnic groups. This pioneering work could inform tailored approaches to improving health outcomes by accounting for cultural practices around cold consumption.

## Supporting information

Wu et al. supplementary material 1 Wu et al. supplementary material

Wu et al. supplementary material 2 Wu et al. supplementary material

## Supplementary material

For supplementary material/s referred to in this article, please visit https://doi.org/10.1017/S000711452510514X
