# Supplementary material for: Cold and hot consumption and health outcomes among US Asian and White populations
Source: Br J Nutr. 2025 Sep 18;134(8):645–59. doi: 10.1017/S000711452510514X (PMC12507458; doi:10.1017/S000711452510514X)
Supplement: Wu et al. supplementary material 1 — Wu et al. supplementary material [file S000711452510514Xsup001.docx]

**Supplemental Figure 1.** Total cold consumption in summer and winter in relation to various health outcomes across different Asian subgroups. Covariates in the multiviable model inlcuded age, body mass index, physical activities, and smoking status.
