# Supplementary material for: Cold and hot consumption and health outcomes among US Asian and White populations
Source: Br J Nutr. 2025 Sep 18;134(8):645–59. doi: 10.1017/S000711452510514X (PMC12507458; doi:10.1017/S000711452510514X)
Supplement: Wu et al. supplementary material 2 — Wu et al. supplementary material [file S000711452510514Xsup002.pdf]

Supplemental Table 1. Cold drinks and meal score among Asian subgroups

	**Chinese** n = 77	**Asian Indian** n = 43	**South Asian** n = 55	**Other Asian** n=37	**P for comparison**
Cold drink>=5-6 times/week in winter, n (%)	17(22%)	13(30%)	19(35%)	14(38%)	**0.007**
Cold meal>=3 times/week in winter, n (%)	10(13%)	14(33%)	11(20%)	8(24%)	**0.01**
Cold drink>=5-6 times/week in summer, n (%)	30(39%)	17(40%)	29(53%)	22(59%)	**0.02**
Cold meal>=3 times/week in summer, n (%)	23(30%)	21(49%)	27(50%)	22(59%)	**0.03**

The total cold drink score summarizes the combined consumption of cold drinks and cold water.

Cold water or drinks refer to the water or beverages that are close to 4°C (iced) or have just been taken out of refrigerators before consumption.

Cold meals were defined as those served below room temperature, such as cold salads, cold sandwiches, sushi, and cold milk.

Bolded results indicate p values ≤0.05 or ≤0.10

Supplemental Table 2. Aging-related subclinical health conditions across different Asian subgroups

	Chinese n = 77	Asian Indian n = 43	South Asian n = 55	Other Asian n = 37	P for comparison
**Mental health**					
Depression score, mean (SD)	2.8	4.6	4.5	3.8	**0.01**
Classified with depression symptoms (score >=5), N (%)	17(22)	18(42)	22(40)	10 (27)	**0.06**
Anxiety score (Mean, SD)	3.2	6.6	5.4	6.0	**0.001**
Classified with anxiety disorder (score>=8), N (%)	7 (9)	16 (37)	20 (36)	5(13)	**0.0001**
Insomnia score, mean (SD)	7.1	7.2	7.7	7.9	0.5
Classified with insomnia symptoms (score>=9), N (%)	19(25)	16(37)	18(33)	16(43)	0.2
**Gut health**					
Often or always with sensation of abdomen fullness, N (%)	14(18)	11(26)	25(46)	15(40)	**0.02**

Abbreviations: SD=standard deviation.

Bolded results indicate p values ≤0.05 or ≤0.10
